# Evaluation of an Internet-Based Behavioral Intervention to Improve Psychosocial Health Outcomes in Children With Insomnia (Better Nights, Better Days): Protocol for a Randomized Controlled Trial

**DOI:** 10.2196/resprot.8348

**Published:** 2018-03-26

**Authors:** Penny V Corkum, Graham J Reid, Wendy A Hall, Roger Godbout, Robyn Stremler, Shelly K Weiss, Reut Gruber, Manisha Witmans, Christine T Chambers, Esmot Ara Begum, Pantelis Andreou, Gabrielle Rigney

**Affiliations:** ^1^ Department of Psychology & Neuroscience Dalhousie University Halifax, NS Canada; ^2^ Department of Psychiatry Dalhousie University Halifax, NS Canada; ^3^ Department of Pediatrics IWK Health Centre Halifax, NS Canada; ^4^ Department of Psychology University of Western Ontario London, ON Canada; ^5^ Department of Family Medicine Schulich School of Medicine and Dentistry University of Western Ontario London, ON Canada; ^6^ Department of Paediatrics Schulich School of Medicine and Dentistry Western University London, ON Canada; ^7^ Children’s Health Research Institute & Lawson Health Research Institute London, ON Canada; ^8^ School of Nursing Faculty of Applied Science University of British Columbia Vancouver, BC Canada; ^9^ Department of Psychiatry Université de Montréal Montréal, QC Canada; ^10^ Lawrence S Bloomberg Faculty of Nursing University of Toronto Toronto, ON Canada; ^11^ Division of Neurology Department of Paediatrics University of Toronto Toronto, ON Canada; ^12^ Department of Psychiatry Faculty of Medicine McGill University Montréal, QC Canada; ^13^ Attention Behavior and Sleep Lab Douglas Mental Health University Institute McGill University Montréal, QC Canada; ^14^ Faculty of Medicine & Dentistry University of Alberta Edmonton, AB Canada; ^15^ Department of Pediatrics Dalhousie University Halifax, NS Canada; ^16^ Centre for Pediatric Pain Research IWK Health Centre Halifax, NS Canada; ^17^ Department of Community Health and Epidemiology Faculty of Medicine Dalhousie University Halifax, NS Canada

**Keywords:** sleep, insomnia, children, randomized controlled trial, eHealth, Internet, treatment

## Abstract

**Background:**

Up to 25% of 1- to 10-year-old children experience insomnia (ie, resisting bedtime, trouble falling asleep, night awakenings, and waking too early in the morning). Insomnia can be associated with excessive daytime sleepiness and negative effects on daytime functioning across multiple domains (eg, behavior, mood, attention, and learning). Despite robust evidence supporting the effectiveness of behavioral treatments for insomnia in children, very few children with insomnia receive these treatments, primarily due to a shortage of available treatment resources.

**Objective:**

The Better Nights, Better Days (BNBD) internet-based program provides a readily accessible electronic health (eHealth) intervention to support parents in providing evidence-based care for insomnia in typically developing children. The purpose of the randomized controlled trial (RCT) is to evaluate the effectiveness of BNBD in treating insomnia in children aged between 1 and 10 years.

**Methods:**

BNBD is a fully automated program, developed based on evidence-based interventions previously tested by the investigators, as well as on the extant literature on this topic. We describe the 2-arm RCT in which participants (500 primary caregivers of children with insomnia residing in Canada) are assigned to intervention or usual care.

**Results:**

The effects of this behavioral sleep eHealth intervention will be assessed at 4 and 8 months postrandomization. Assessment includes both sleep (actigraphy, sleep diary) and daytime functioning of the children and daytime functioning of their parents. Results will be reported using the standards set out in the Consolidated Standards of Reporting Trials statement.

**Conclusions:**

If the intervention is supported by the results of the RCT, we plan to commercialize this program so that it is sustainable and available at a low cost to all families with internet access.

**Trial Registration:**

ClinicalTrials.gov NCT02243501; https://clinicaltrials.gov/show/NCT02243501 (Archived by WebCite at http://www.webcitation.org/6x8Z5pBui)

## Introduction

### Pediatric Sleep Problems and Daytime Consequences

Sleep problems are highly prevalent among children [1,2]. Although there is a range of physiological causes for inadequate sleep (eg, sleep apnea, restless legs syndrome, and narcolepsy), by far the most common problem is *insomnia*. This condition is defined as “repeated difficulty with sleep initiation, duration, consolidation, or quality that occurs despite age appropriate time and opportunity for sleep, which results in some form of daytime functional impairment for the child and/or family” [3]. Insomnia affects approximately 10% to 25% of typically developing children in the general population. The higher estimates include children showing bedtime resistance; this is probably the most relevant rate, because this symptom is independently associated with functional impairment for the child and primary caregiver, henceforth referred to as parent. Sleep habits developed in childhood shape sleep habits in adulthood [4]; therefore, children with sleep problems often grow into adolescents and adults with sleep problems [5]. Early intervention therefore holds the promise of interrupting the cycle of insomnia that plagues many individuals throughout their lives.

Although there is less research on the effects of sleep problems in children than adults, there is evidence that poor sleep in children is associated with deficits in cognitive functioning (eg, attention) as well as with poor emotional regulation, lower academic achievement, and increases in negative mood and behavioral problems [6-11]. There is also preliminary evidence that short sleep in young children may be related to poorer physical health, such as increased obesity [12]. There is further evidence that sleep problems in children are associated with impaired parental sleep and daytime functioning, including increased parental stress, punitive parenting, and poorer psychological functioning in parents [13-17]. Most studies of the impact of children’s poor sleep have been correlational in nature, rather than based on experimental manipulations. A national survey of 1273 members of the American Academy of Child and Adolescent Psychiatry outlining the prevalence of insomnia in pediatric populations in clinical practice speaks to the need for further experimental research to evaluate the impact of children’s sleep problems and available treatment [18].

### Treatment of Pediatric Insomnia

The most common treatment for insomnia in children (and adults) is medication. A chart review in outpatient health centers found that 81% of children presenting with a sleep disorder were prescribed medications [19]. This pattern of care is troubling because there are no approved medications for insomnia in children, and there are concerns about the safety and side effects of these medications [20,21]. Moreover, these medications are not effective in the long term, and their use remains unjustified in most children [22]. In contrast, behavioral interventions were recommended in only 22% of cases [19], despite overwhelming evidence of their safety, short- and long-term effectiveness [3,9,23-27], and acceptance by parents [28,29]. A systematic literature review of behavioral intervention studies reported that 94% of included studies (49/52) found that these interventions were effective in treating insomnia, and 80% of children treated demonstrated significantly improved sleep [3]. Behavioral interventions not only have a direct impact on children but also improve parents’ sleep, and increase their sense of competence and control and ability to cope [30,31], as well as change their knowledge and perceptions about sleep problems [6]. The available evidence convincingly demonstrates that behavioral interventions should be the first line of treatment, but this is typically not the case.

### Treatment Barriers

The primary reasons for the low rate of treatment are due to a shortage of available treatment resources and lack of knowledge among health professionals about sleep disorders and treatment [32]. Together, these issues result in sleep problems in children often being minimized or ignored [33,34], and being inadequately addressed even when acknowledged. Evidence-based behavioral intervention protocols are not readily available for clinical use, which is a classic case of a failure in knowledge translation. Even when effective treatments are available, they are usually provided in a traditional service delivery framework, involving weekly parent training sessions [35-38] or multiple clinic visits with health care professionals [39,40]. These traditional approaches may be difficult for parents because of scheduling conflicts, incidental costs, and travel difficulties. Thus, a major challenge to the delivery of behavioral treatments is the inability of many families (especially those in rural or remote areas or those who are economically disadvantaged and lack insurance coverage for psychological services) to access the services they require [41].

### Internet-Based Treatment Programs

A growing body of research supports the effectiveness of interventions delivered via the internet (eHealth, electronic health), with a number of randomized controlled trials (RCTs) demonstrating effectiveness for a range of chronic health and mental health disorders in adults and children [42-44]. There have been several studies evaluating internet-based treatment programs for adult insomnia [45-50]. A systematic review and meta-analysis published in 2016 on the effectiveness of internet-delivered treatment for insomnia in adults, which included 11 RCTs, reported that there was significant improvement in sleep parameters among the participants who received eHealth interventions [51]. A second systematic and meta-analysis published in 2016, which included 15 RCTs, also reported improved sleep in adults who received internet-based behavioral interventions [52]. Most recently, a systematic review examining mobile phone interventions for sleep disorders and quality published in 2017 identified 12 RCTs that determined that mobile interventions support the capability of attenuating sleep disorders and enhancing sleep quality [53]. Although internet-delivered interventions have proven effective for adults, to date, the effectiveness of eHealth interventions has not been established for typically developing children. To our knowledge, there are no published reports evaluating internet-based interventions for insomnia in preschool- and school-aged children. A recent study described the development of a mobile phone app to deliver cognitive behavioral therapy for insomnia in adolescents [54]. There are 2 studies that have reported on an internet-delivered intervention for infants and toddlers [55,56], both of which found significant improvement in children’s sleep including decreased sleep onset latency, decreased frequency and duration of night awakening, and significant improvement in parental sleep and mood as well as increased parental confidence to manage children’s sleep. Given that a survey of parents of young children found that all parents indicated an interest in internet-based treatment programs for sleep problems [57] and that 82% of North American parents with children ages 6 to 16 years old have internet access [58], the internet has the potential to be a powerful tool to overcome barriers to the delivery of treatment for pediatric insomnia.

### Rationale

The Better Nights, Better Days (BNBD) program, which is an eHealth intervention for primary caregivers of children ages 1 to 10 years who present with insomnia, can bridge this knowledge to practice gap by providing a potential solution to one of the most common treatment barriers—access to care. BNBD is an innovative, bilingual (English and French) eHealth program for parents, which aims to provide accessible and evidence-informed care for insomnia in typically developing children.

This paper presents the protocol for a planned RCT to evaluate the effectiveness of the BNBD intervention for the treatment of insomnia in children. This is a 2-arm design, using an equal allocation ratio of 1:1, comparing participants assigned to the intervention group who receive the BNBD intervention (treatment) and those assigned to the usual care group who do not receive the BNBD intervention and are able to access other treatment resources (control). Assessments are conducted at 3 periods: baseline (pretreatment), 4 months postrandomization (end of treatment), and 8 months postrandomization (follow-up). To assess the impact of the intervention, the primary outcome measure of sleep efficiency is evaluated using data collected from actigraphs worn by children and sleep diaries completed by parents. Sleep efficiency is the ratio of total time spent asleep to the total amount of time spent in bed. In addition, secondary outcomes are captured by questionnaires that are collected to evaluate children’s sleep and psychosocial health, as well as parental daytime functioning and psychosocial health outcomes.

### Study Objectives

The purpose of the trial is to evaluate the effectiveness of BNBD, an eHealth intervention for insomnia in children 1 to 10 years of age.

#### Primary Objective and Hypothesis

The primary objective is to assess the *immediate impact* (baseline vs 4 months) of the intervention on children’s sleep.

We hypothesize that at the end of the 4-month assessment period, children randomized to the intervention group will evidence improvement in their symptoms of insomnia compared with children in the usual care group. The outcome variables are sleep efficiency collected by both an objective measure (actigraphy) and a parent report measure (sleep diary).

There are 2 hypotheses for the primary outcome:

1. We hypothesize that children in the intervention group will show improvements in sleep efficiency calculated using *actigraphy* data compared with the usual care group.
2. We hypothesize that children in the intervention group will show improvements in sleep efficiency calculated using *sleep diary* data compared with the usual care group.

Note that sleep efficiency is a measure that captures both sleep quantity and quality and is defined as the amount of total sleep time divided by the amount of time spent in bed with the goal to be sleeping. For example, if a child went to bed at 8 PM and woke at 8 AM, but took 1 hour to fall asleep and was awake for 30 min throughout the night, the child’s sleep efficiency would be 87.5% (ie, (630 min/720 min) × 100).

#### Secondary Objectives and Hypothesis

The secondary objectives are to (1) evaluate the longer-term impact (baseline vs 8 months) on children’s sleep and psychosocial health and (2) examine the impact on parent daytime fatigue and psychosocial health outcomes.

1. We hypothesize that *children* in the intervention group will show improvement compared with children in the usual care group at the 8-month follow-up in their symptoms of insomnia, based on improvements in sleep efficiency calculated using actigraphy and sleep diary.
2. We hypothesize that *children* in the intervention group will show improvement compared with children in the usual care group at the 8-month follow-up in their symptoms of insomnia based on questionnaires that capture symptoms of insomnia.
3. We hypothesize that *children* in the intervention group will show improvement compared with children in the usual care group, in their psychosocial health at the 8-month follow-up, evaluated using a questionnaire that identifies internalizing and externalizing behaviors and quality of life of children.
4. We hypothesize that *parents* randomized to the intervention group, when compared with parents randomized to the usual care group, will show (1) decreased daytime fatigue, (2) increased psychosocial health, and (3) improved parenting strategies at the 8-month follow-up based on questionnaires.

## Methods

### Study Design

The study is a 2-arm RCT, using 1-to-1 allocation, comparing participants assigned to receive either the BNBD intervention (intervention group) or the control group (usual care group). The usual care group will receive the intervention at the end of the 8-month follow-up assessment. The Consolidated Standards of Reporting Trials (CONSORT) 2010 [59,60] guidelines and CONSORT eHealth guidelines [61] were used to design the trial and will be adhered to when reporting the results of the trial. The intervention is being delivered across Canada. The study is coordinated through Dr Corkum’s research laboratory (Corkum LABS; Learning, Attention, Behaviour and Sleep) at Dalhousie University. Both the intervention group and usual care group are able to access any resources or programs and services while enrolled in the study, if they so choose.

### Subject Population

We plan to enroll a total of 500 participants in the study. Participants are being recruited from across all Canadian provinces and territories.

#### Inclusion Criteria

Potential participants must meet the following criteria to be eligible to participate in the study:

- Primary caregiver of a child aged 1 to 10 years. (Note that children younger than 12 months are not targeted because sleep patterns are still being established; youth over 10 years of age may be entering puberty, during which time other sleep problems can arise. Moreover, children over 10 years of age may have more control over their own sleep patterns and as such should be included in an intervention. This intervention is only delivered to parents.)
- Live in any province or territory in Canada.
- Have regular access to high-speed internet connection and an email account.
- Comfortable communicating in English or French for day-to-day tasks (eg, listening to the news on the radio, watching TV, and reading books, magazines).
- Child has insomnia, defined as having sleep onset disturbance based on the criteria established by Anders and Dahl [62].

#### Exclusion Criteria

Potential participants who meet any of the following criteria are not eligible to participate in the study:

- Parent wishes to “bed-share” with their child [63].
- Child has a probable intrinsic sleep disorder (eg, sleep apnea) as assessed based on a questionnaire that screens for pediatric sleep-related breathing disorders [64].
- Child has a significant medical disorder that interferes with sleep (eg, asthma attacks during night, tube feeding, nonambulatory, and severe developmental disability affecting sensory systems such as vision), as determined by parent report and expert clinical review based on an author-made health-related questionnaire.
- Child has a mental health disorder that has required hospitalization or residential care or current use of psychotropic medications that are known to interfere with sleep (eg, stimulant medication for attention-deficit/ hyperactivity disorder), as determined by parent report and expert clinical review based on an author-made health-related questionnaire.
- Parent does not have appropriate level of English or French language skill to engage in this intervention, assessed based on a questionnaire that captures proficiency in communication [65].

### Recruitment

The recruitment strategy is a multiprong approach across Canada targeting 3 groups: (1) parents, (2) external stakeholders and organizations (ie, our partners including health care provider associations), and (3) the media.

The target audience of the BNBD intervention is parents with regular access to the internet; therefore, the recruitment strategy is primarily grounded on internet-based tools, notably the study website [66] and social media, including Facebook, Twitter, Instagram, and Pinterest. However, traditional recruitment methods are also employed, including media interviews and printed posters and conference presentations, to reach both potential participants and the health care community.

We plan to enroll 500 eligible participants in this study, with 80.0% (400/500) of the sample English speaking and 20.0% (100/500) of the sample French speaking, consistent with the Canadian demographics. The study sample is also stratified on the age group of the participant’s child (toddler: 1-2 years; preschool: 3-5 years; and school-aged: 6-10 years), with equal numbers of participants enrolled from each of the 3 categories (target n=167).

**Biopsychosocial model of sleep.**

Bio

- Opponent process model
- Physiological arousal

Psycho

- Classical conditioning
- Operant conditioning
- Psychological arousal
- Emotional regulation

Social

- Dyadic processes
- Family processes
- Environmental, cultural, socioeconomic influences

Recruitment is targeted to ensure that the study population includes representation from across Canada. Canada is divided into 4 geographical regions: Atlantic (New Brunswick, Newfoundland and Labrador, Nova Scotia, Prince Edward Island,), Central (Ontario, Quebec), Prairies and Northern Territories (Alberta, Manitoba, Saskatchewan, Northwest Territories, Nunavut, Yukon), and the West Coast (British Columbia).

### Behavioral Change Model

The overarching model is based on Ritterband’s eHealth behavioral change model [67]. This model states that an effective internet-based intervention produces and maintains behavior change and symptom improvement through the context (including user characteristics, environment, and website characteristics), which in turn impacts behavior change mechanisms, and ultimately impacts outcomes. In this situation, the behavior change mechanism is focused on changing the parents’ cognitions, affective responses, and behavior responses, to change the child’s insomnia symptoms. We used a biopsychosocial model to conceptualize the multiple predisposing, precipitating, and perpetuating factors related to the development and maintenance of, and the treatment of, insomnia in children. See Textbox 1 for further details.

### Electronic Health (eHealth) Intervention

Participants randomized to the intervention group receive access to the BNBD intervention immediately, whereas participants randomized to the usual care condition receive access after they complete the 8-month follow-up assessment.

The BNBD intervention is fully self-guided (ie, there is no contact with coaches or clinicians), empowering parents to implement strategies independently. The program introduces evidence-based interventions, tailored content (ie, participants create personalized sleep routines, set individualized goals, and receive custom feedback on progress), and age-specific information delivered primarily through videos, supported by graphics, animations, and interactive elements to engage and encourage parents. Access to built-in tools and supports, such as sleep diaries and goal setting and tracking, provides feedback on participants’ progress.

The intervention includes 5 sessions made available sequentially to participants. Table 1 summarizes the content of the intervention. The recommended completion time of the intervention ranges from 5 to 10 weeks. Participants receive an automated email reminder when a session becomes available, a prompt to complete each session, and 7 notices that the recommended completion time is approaching. The core content is delivered primarily via videos and interactive activities to support the parents’ learning of these new skills.

Each session provides evidence-based information to parents, strategies for implementation of best practices to address sleep problems, and access to additional help and advice through a “Roadblock” question-and-answer style support detailing common obstacles to implementing the recommended interventional strategies and evidence-based methods to navigate these obstacles. There is also a “Reward Center” where parents can develop reward programs (eg, sticker charts) to help support the implementation of these sleep intervention strategies.

The program can be used at a time that is convenient for the participants, removing barriers to care and providing services in the comfort and privacy of their own homes. The intervention is hosted on the BeHealth Solution, LLC’s proprietary technology platform [68] and can be accessed through participants’ desktops, laptops, tablets, and mobile phones.

Before beginning each session, starting with Session 2, participants are asked whether they wish to continue the intervention program. If participants indicate they are not interested in participating further, they are asked to complete a questionnaire and provide the reason, in an open-ended question, regarding why they no longer wish to continue the program.

**Table 1:** Intervention sessions.

Session	Topic overview
Sleep information	Characteristics of sleep; types of sleep problems, sleep need; how sleep problems develop; impact and treatment of sleep problems
Healthy sleep practices	Daytime and bedtime routines; sleep hygiene/healthy sleep practices; sleep scheduling (including napping) and sleep routines
Independent settling at bedtime	Settling at bedtime; parents choose a sleep intervention that best fits their needs from 3 intervention strategies: controlled comforting, camping out, and bedtime fading
Night waking, napping, and early morning awakenings	Applying strategies to night waking; applying strategies to early morning awakenings; applying strategies to napping
Looking back and ahead	Relapse prevention; looking back at goals and progress; common pitfalls/roadblocks; what to expect at new developmental milestones; dealing with other sleep problems; making a plan

This allows participants the freedom to discontinue the program at any time and allows us to assess any issues that contributed to discontinuation. To allow for a process-level analysis, before starting each session, participants are also asked to complete the Insomnia Severity Index [69] scale and rate the sleep quality of their child to evaluate pediatric insomnia symptoms.

In addition, 2 measures are used to assess treatment fidelity. First, parents’ implementation of the intervention is assessed using process measures administered at the beginning of each session of the program. In advance of each subsequent module, participants are asked to record how carefully they reviewed the material, what percentage of the recommended strategies they implemented, and how successful they were in implementing these strategies, as well as to provide an estimate of the overall percentage improvement of their child’s sleep problems. Second, computer-generated user statistics captured by the intervention platform software are used to assess adherence, such as the number of times the site was accessed and the period of time taken to complete each module.

### Usability and Quality Assurance

A usability study took place from September 2013 to January 2014 to evaluate user performance and satisfaction with the BNBD intervention. The study [70] was conducted with a prototype of the BNBD intervention, and both qualitative and quantitative data were collected. Qualitative data were analyzed using Ritterband’s eHealth model [67] to better understand how any potential barriers to intervention use (eg, level of ease of accessing the internet) could be corrected and addressed (eg, increased social presence, increased interactivity and personalization). The results of this usability study were used to revise the BNBD intervention.

Quality assurance was also undertaken to evaluate the functionality of the intervention platform in the spring of 2016. Internal reviewers, including program developers with BeHealth Solutions, LLC, and research staff at Corkum LABS reviewed the intervention to identify technical problems. In addition to this, 6 parents of children aged 1 to 10 years old served as external reviewers for the program and completed 5 questionnaires to provide structured feedback on the technical operation of each session of the intervention. The goal of this step was to identify functional problems with the program (eg, broken links, incorrect routing) before conducting the RCT.

### Measures

#### Primary Outcome Measures

The primary outcome variable is sleep efficiency based on both actigraphy and parent report sleep diary data. Actigraphs are couriered to the participants and sleep diary entries are completed on the internet by the parents. Participants are asked to fill out a sleep diary for 7 consecutive days, and the child is asked to wear an actigraph during the same time period. The dates of collection of actigraphy data should correspond to the sleep diary data collection dates. Sleep diary and actigraphy data are collected at each assessment point.

##### Actigraphy

An actigraph is a battery-operated device utilizing an accelerometer to detect motor activity. We use the Philips Respironics Actiwatch 2 (Koninklijke Philips N.V., Amsterdam, NL). When an actigraph is worn, a computer chip located inside the device records movement, which is used to determine a number of sleep variables, including but not limited to sleep efficiency (captured from lights out to awakening), sleep onset, total sleep time, and night waking [9,18].

Participants are asked to have their child wear the actigraph on their child’s wrist of their less dominate hand. Children 1 to 2 years of age wear the actigraph on their ankle. Participants are instructed to record any instances in which the actigraph is removed from their child’s body for any length of time. Participants are instructed to have their child wear the actigraph for 7 days, with a minimum requirement of 5 days.

##### Sleep Diary

An internet-based sleep diary was developed based on systematic reviews by Meltzer and Mindell [24] and Wu and colleagues [71]. Sleep diaries have been validated against actigraphy and polysomnography and have demonstrated good face validity and high internal consistency when used with child participants [72]. The sleep diary requires approximately 10 min to complete. The internet-based sleep diary is housed in the Research Electronic Data Capture (REDCap) platform (Vanderbilt University, Nashville, US), a secure, electronic, data capture system [73]. The internet-based sleep diary underwent technical and usability testing by research staff and external reviewers, as well as 6 parents of children aged 1 to 10 years, to ensure the proper functionality of the measure. Parents can either complete the sleep diary directly online or print a copy and enter the information at a later point (within a 3-day time window). Data entered by participants into the internet-based sleep diary entry form are automatically populated into the REDCap database.

The sleep diary contains 25 items measuring the following variables: sleep duration, nighttime sleep duration, daytime sleep duration, sleep onset latency, bedtime, wake time, presence and frequency of night awakening, and the presence and frequency of bedtime resistance. The sleep diary also provides a measure of time spent in bed extracted from the time the light was turned off (“Down for the night”) to the time light was turned on (“Up for the day”). Sleep efficiency is calculated from these variables.

#### Secondary Outcome Measures and Measures for Exploratory Analyses

Secondary outcomes and exploratory outcomes are assessed through the administration of internet-based questionnaires to assess children’s sleep and psychosocial health, parental function, treatment barriers, and predictors to successful intervention adherence. Each participant answers 13 to 15 questionnaires based on the group assignment (intervention or usual care) and age of the child.

All measures are available in both English and French, based on whether the participant has enrolled in the English or French language trial. All secondary and exploratory outcome measures are administered on the internet via REDCap. All electronic questionnaires underwent technical and usability testing by research staff and external reviewers, as well as by 6 parents of children ages 1-10 years. All questionnaires are displayed in a sequential manner. Certain measures are age-dependent, and REDCap automatically delivers them accordingly. All measures, and the time points that each is administered, are indicated in Table 2.

Please see Multimedia Appendix 1 for a detailed description of all measures used throughout the study.

### Study Procedures

**Figure 1:**
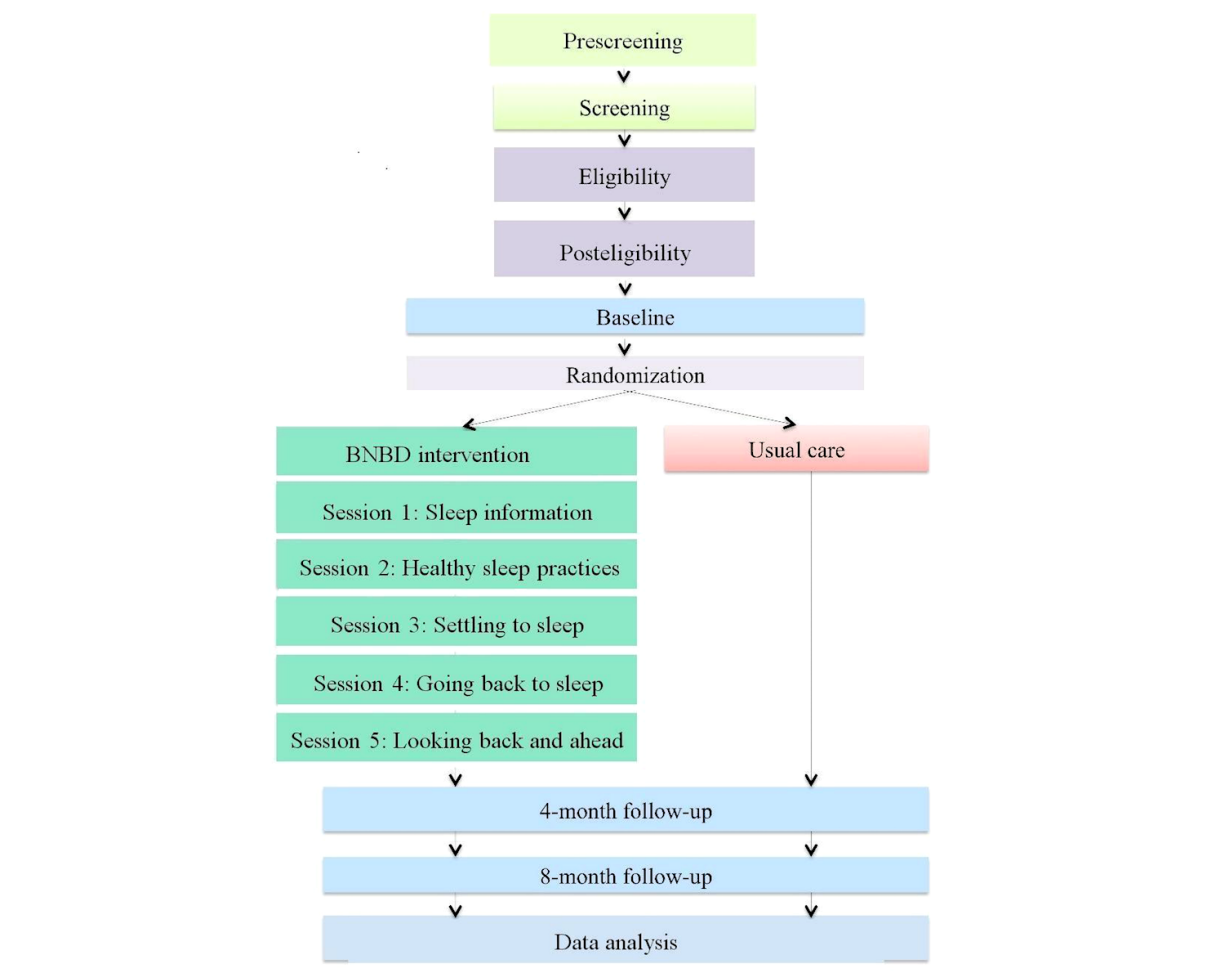
Schematic overview of the study.

Figure 1 displays the schematic overview of the study and each step is described below.

#### Prescreening

During the prescreening process, potential participants conduct a self-screening on the BNBD website [66]. Here, interested individuals read the inclusion and exclusion criteria in lay terms and self-assess to determine if they may be eligible. If parents feel they may meet eligibility criteria and are interested in the study, they click a link directing them to the REDCap database to continue with screening. A screening information and consent form to participate in the screening process is presented, and potential participants must consent to proceed. Participants are given an opportunity to contact research staff by email to have any questions answered and concerns addressed, if they so choose.

#### Screening and Consent

After completing the screening information and consent form, participants complete the screening questionnaire to assess whether they meet inclusion criteria [63]. Participant responses to the screening questionnaire are automatically assessed in REDCap using predefined eligibility criteria, and participants immediately receive an onscreen message stating whether they are eligible or ineligible to continue. Individuals who are eligible at screening are invited to consent to participate in the full study by completing the information and consent form. The information and consent form and study data are housed in different databases. Individuals who are ineligible are informed that they do not meet the study requirements. Participants are given an opportunity to contact research staff by email to have any questions answered and concerns addressed.

#### Eligibility

After completing the information and consent form, participants complete 4 eligibility questionnaires: behavioral insomnia questionnaire [62], pediatric sleep questionnaire [64], health-related questionnaire (HRQ; author made), and single item literacy scale [65]. REDCap automatically scores these questionnaires and determines if the participant is eligible or if further review is required. Further review is required if the participant responds “yes” to any of the questions on the HRQ.

The HRQ is reviewed within 72 hours post completion by a subcommittee comprised of 3 coinvestigators including at least 1 psychologist and 1 physician (also trained in sleep medicine) to evaluate if the child has any significant physical health disorders, mental health disorders, or sleep disorders that would make them ineligible to participate.

Participants are provided with the email address of the research staff whom they may contact in the event that they have concerns or questions about the decisions related to eligibility. Participants may also request a phone call to speak with research staff about these decisions.

#### Posteligibility

Eligible participants provide their mailing address and telephone number on the identifying information questionnaire, collected in a separate REDCap database (to ensure the privacy of participants' personal information). Research staff members contact participants by telephone to confirm their mailing address and answer questions regarding study procedures. Once contact with the participant is made, the study package, including the actigraph and sleep diary, is couriered to the participant’s preferred address.

#### Baseline

Once participants receive their study package, an automated invitation email from REDCap is delivered to the participant to commence baseline measures. Participants complete 7 days of sleep diary entries and collect 7 days of actigraphy data. Participants also complete a series of questionnaires on REDCap before the end of the 7-day actigraphy and sleep diary data collection.

**Table 2:** Measures and collection schedule throughout the study period. The X symbol denotes at which assessment period(s) participants complete each measure.

Measures			Screening	Eligibility	Posteligibility	Baseline	Follow-up at 4 months	Follow-up at 8 months		
**Screening measures**										
	Screening questionnaire [63]		X							
**Eligibility measures**										
	Behavioral insomnia questionnaire [62,74]			X		X^a^	X	X		
	Pediatric sleep questionnaire [64,74]			X						
	Health-related questionnaire			X						
	Single item literacy screen [65]			X						
**Posteligibility measures**										
	Identifying information questionnaire				X					
**Outcome measures**										
	Demographic questionnaire [75]					X				
	**Child sleep/insomnia**									
		Actigraphy				X	X	X		
		Sleep diary				X	X	X		
		Tayside children’s sleep questionnaire/sleep disturbance scale for children [74,76,77]				X	X	X		
	**Child daytime functioning—psychosocial/physical health**									
		Pediatric quality of life [78,79]				X	X	X		
		Child behavior checklist for ages 1½ to 5 years/child behavior checklist [80,81,82,83]				X	X	X		
		Caregiver-teacher report form/teacher report form [80,82,83]				X	X	X		
	**Parent functioning/psychosocial health**									
		Single item fatigue impact scale [84,85]				X	X	X		
		Depression, anxiety and stress scales [86-89]				X	X	X		
		Parenting scale [90,91]				X	X	X		
**Measures for exploratory analyses**										
	Children’s physical activity index [92]					X		X		
	Body mass index [93-95]					X		X		
	Parent’s rating of clinically significant improvement [96]					X	X	X		
	Treatment utilization questionnaire [97]					X	X	X		
	Willingness to pay					X	X^b^			
	Barriers to treatment participation scale [98,99]						X^b^			
	Client satisfaction questionnaire [100,101]						X^b^			
	Readiness for change [102-104]					X				
	Bedtime routines questionnaire [105]					X	X			

^a^If baseline assessment is >30 days from eligibility assessment, the behavioral insomnia questionnaire is repeated at baseline.

^b^Intervention arm only.

#### Randomization

After completion of baseline measures, participants are randomized. A blocked stratified randomization is used with 1-to-1 allocation for parents to either the intervention group or the usual care group. Participants are not blinded to their assignment group. Participants are stratified by age groups: toddler (1-2 years of age); preschool-aged (3-5 years of age); and school-aged (6-10 years of age). Blocks of 4 are used to randomize the participants for each stratum (ie, each age group).

The randomization table was created using the block random function in the R statistical software [106] and inputted into REDCap by a research staff member who is not associated with the management of participants. The only individuals who have access to the randomization table are not associated with participant management and include the research associate (EAB) who created the random allocation table based on the study design and a coinvestigator with extensive RCT design experience (RS) and the study statistician (PA), who reviewed the randomization table. The statistician conducting the primary analysis will be blinded to participant allocation to intervention or usual care groups (ie, the statistician will not be given the code for this variable).

Research staff members randomize participants using an automated REDCap procedure. The randomization table, created in Microsoft Excel, is uploaded into the REDCap database, where it cannot be modified or accessed. Only the research associate is able to view the randomization table. Once a participant completes baseline assessment, research staff members confirm all criteria are met and “trigger” the automated randomization by clicking a button within the program. An automated email is sent to participants to notify them of the randomization result.

#### Follow-Up at 4 Months and 8 Months

Primary outcome measures (sleep diary and actigraphy), secondary outcome measures (questionnaires), and measures to address exploratory questions are administered to all participants at baseline and at 4 and 8 months postrandomization. Participants receive an automated invitation email from REDCap to start their 4-month follow-up after 16 weeks postrandomization, and to start their 8-month follow-up 32 weeks postrandomization.

If participants do not begin follow-up assessment, research staff members make 12 attempts to contact participants by email and telephone at different times of day (eg, afternoon, evening) and various days of the week (eg, weekday, weekend), over the course 6 weeks (from 14 weeks postrandomization to 20 weeks postrandomization at 4-month follow-up, and 30 weeks to 36 weeks postrandomization at 8-month follow-up). This procedure is designed to maintain communication with participants and ensure that outcome data are collected. Participants are deemed lost to follow-up for that assessment period if 4-month and 8-month assessments are incomplete by 20 weeks or 36 weeks postrandomization, respectively.

#### Compensation

To compensate participants for their time and to maximize adherence rates, participants receive an honorarium of $25 CAD for the completion of all measures at each of the 3 collection time points, as well as a completion stipend of $25 CAD at the end of the 8-month follow-up, if they completed all 3 assessments. Therefore, a maximum of $100 CAD per participant is given as an honorarium for study participation. At study completion, participants are entered into a draw to win a tablet; one entry is generated for each day that the participant completed the sleep diary and has corresponding actigraphy data.

### Statistical Analysis

#### Sample Size Analysis

A total goal of 500 consented participants is expected. Using the assumption that 50.0% (250/500) of participants will be lost to follow-up, the target sample size at the end of the study is n=250 [107-109]. This gives us power of .80 to detect a significant group-by-time interaction with the effect size (*d*)=0.45 and alpha set to .025, for primary outcome measures calculated from actigraphy and sleep diary from baseline to 4-month follow-up. Given that 2 primary outcome variables are being used (sleep efficiency from actigraphy and sleep efficiency from sleep diaries), alpha was divided by 2 so that *P*=.025, rather than *P*=.05 would be accepted as indication of statistical significance. This is based on the assumption that an effect size of *d*=0.45 is clinically significant for sleep efficiency as measured by actigraphy and sleep diary [45].

#### Data Analysis

The primary outcome variables that will be used to evaluate the impact of the intervention program on insomnia symptoms are sleep efficiency from actigraphy and sleep efficiency from sleep diary data. Overall, 2 primary outcome measures are included to capture these variables of interest using both an objective measure of sleep (ie, actigraphy) and a parent report/subjective measure of sleep (ie, sleep diary), thus allowing us to compare our results with existing research in the field. In unblinded RCTs (ie, in which participants know their group assignment such as this study), there is evidence of bias toward significance of the active treatment; thus, an objective measure (eg, actigraphy) is used to mitigate this bias [110]. However, as parents are the primary decision makers with respect to seeking treatment for their child’s health problems, it is also critical to obtain their perspective. Further, inclusion of patient-reported outcomes is recommended when testing interventions [111].

Secondary outcomes for this study examine the maintenance of changes in sleep, as well as the impact on the child’s psychosocial health (behavioral, attentional, and emotional functioning) and on parents’ psychosocial health (ie, psychological adjustment). Data analyses are overseen by the research team, including the statistician, using an intent-to-treat data analytic approach [112].

Conditional growth model methodology, also known as hierarchical models, a generalization of the standard linear model, which permits data to exhibit correlation and nonconstant variability, will be used to fit each outcome at baseline, 4 months, and 8 months [113] using PROC MIXED in the Statistical Analysis System. Model formulation is at 2 levels, with participants as level 1, and treatment group as level 2. The level 1 model is a linear individual growth model, and the level 2 model expresses variation in parameters from the growth model as random effects unrelated to any person-level covariates. For each parent, the actual time (in days) from baseline to when each follow-up assessment is completed is also entered, to account for variation in when parents actually complete assessments.

##### Primary Outcome

For the primary outcome (sleep efficiency based on actigraphy and sleep diary data), we will test for a significant interaction effect between time and group, which would indicate differential rate of change over time between groups. Differences in the estimated means between the intervention group and usual care group will also be compared at the 4-month post treatment time point (primary end point) to determine the magnitude of differences between groups [114].

##### Secondary Outcome

For the secondary outcomes, we will test differences between the 2 groups modeling changes in the outcome variables across the 3 points of measurement (baseline, 4 months, and 8 months postrandomization) to determine treatment effects at 8 months postrandomization. Again, a significant time × group interaction would indicate differential rate of change over time between groups. Differences in the estimated means at the 4-month and 8-month posttreatment time points will also be examined.

To examine the possibility of differential response to treatment across the 3 age groups, we will use a growth curve modeling of the 2 primary outcomes (sleep diary and actigraphy). Analysis is based on the methodology of the extension of the generalized linear model for longitudinal data, namely marginal and random effects modeling, which allows for varying intercepts and slopes across subjects, “G-side” random effects, and different within-person error variance covariance structure, “R-side” random effects [115,116]. An interaction of age group (toddler, preschool-aged, and school-aged) × time (3 assessments) × randomization group (intervention and usual care) would indicate differential response to treatment.

An intent-to-treat analysis will be conducted. Multiple imputation techniques will be used to deal with missing data. This method works well on longitudinal data and is robust to violations of non-normality of the variables used in the analysis.

#### Clinical Significance

The clinical significance of an improvement in outcomes in response to treatment for pediatric sleep problems has not been defined. Various methods have been used to examine clinical significance [117,118]. Minimal important difference (MID) is an important metric of clinical significance. The MID refers to “the smallest difference in score in the domain of interest which patients perceive as beneficial and which would mandate, in the absence of troublesome side effects and excessive cost, a change in the patient’s (health care) management” [119]. The MID has not been determined for most pediatric health issues utilizing parent-reported outcomes [120]. We will examine clinical significance in 2 ways for both of the primary outcomes. First, the reliable change index (RCI) will be computed and the percentage of cases that exceeded RCI>1.96 will be determined [121]. The RCI is a distribution-based metric that informs the extent to which an observed change is reliable; it is often used in mental-health treatment studies [122,123]. Second, at baseline, all participants are asked to rate the “smallest amount of improvement” on their child’s sleep problems that they would be satisfied with, using a 10 cm visual analog scale (not at all improved, 0%; completely improved, 100%). The average is used as a method of estimating the MID [124] and has been used in other pediatric sleep intervention trials [125]. The percentage of cases that achieve at least this level of improvement between baseline and either 4 months or 8 months postrandomization will be computed.

## Results

The RCT for the English language population was launched in September 2016 and the expected date of completion is February 2018. The RCT of the French language population was launched in May 2017 and is expected to be completed in February 2018. Data analysis will be completed by 2019.

## Discussion

### Main Goals

Our primary purpose for this study is to provide a readily accessible, evidence-based eHealth intervention to increase access to care for insomnia in typically developing children aged 1 to 10 years. With the significant percentage of the Canadian population with internet access [126], eHealth delivery has the capacity to be a powerful tool to overcome traditional systemic barriers to treatment access. The internet-based BNBD program similarly has the potential to (1) be integrated within a stepped-care model of pediatric services, (2) improve parents’ knowledge about behavioral treatments for insomnia, (3) improve accessibility of treatment interventions for children and their families, and (4) reduce service demands at the frontline of clinical practice.

### Ethical Considerations

#### Informed Consent

Ethics approval was granted by IWK Health Centre Research Ethics Board. To ensure informed consent, participants must sign the internet-based screening information and consent form and the information and consent form after the nature of the study has been fully explained. Participants are not able to proceed to any study-related activity before electronically signing consent. These consent forms inform participants that participation is voluntary and that they may withdraw consent to participate at any time, and they are informed of the aims, methods, benefits, and risks of the study. All participants are given an opportunity to contact research staff by email or to request a phone call to have any questions answered and concerns addressed. A series of true and false questions to test participants’ knowledge of their research rights are used to ensure informed consent. A copy of both the screening information and consent form and the information and consent form are available to be downloaded and saved by participants. Participants provide an electronic signature and date and click an “I Agree” button.

#### Privacy, Personal Health Information, and Study Data

The study is conducted in accordance with all applicable standards and procedures of the IWK Health Centre, as well as the Personal Health Information Act, Personal Information International Disclosure Protection Act, and the Tri-Council Policy Statement: Ethical Conduct for Research Involving Humans.

Personal health information and study data that are collected, used, or disclosed are limited to those data that are necessary to fulfill the objectives of the study explained in this protocol, and all data are handled in a confidential manner. Study data are identified using a unique identifier code. A key file is used to link this identifier to participants’ email addresses. Data are not affiliated with any personal health information, and all data are collected and stored on the Canadian-based secure servers, the REDCap electronic data capture system and the Dalhousie University server. Identifying information is accessible only to the study investigators, postdoctoral fellow, and data management and research staff. All study data will be kept in a secure and confidential location for at least 5 years post data publication and then destroyed according to IWK Health Centre’s policy.

#### Safety Monitoring

This behavioral intervention involves minimal risk to the participants. Participants are provided with the email address of research staff whom they may contact in the event that they have concerns or questions related to the study. The study safety-monitoring plan involves communication between the research staff, who are in contact with participants, and the principal investigator (PVC), a registered psychologist. Any difficulties experienced by the participants that cannot be dealt with adequately by research staff are immediately communicated to the principal investigator or her delegate (coinvestigator) and follow-up with the participant is made to ensure their well-being. Data monitoring for adverse events or trends in outcome has not been undertaken given that this is a low-risk study. An adverse events committee, composed of the 3 members from Dalhousie University, not associated with the BNBD study, and having medical, psychology, and sleep expertise, will review adverse events if one becomes known during the course of the study.

### Commercialization

If the BNBD intervention is found to be effective, to ensure its sustainability, the investigators anticipate commercialization of the BNBD intervention. The investigators will work to commercialize this program for a reasonable rate, so that all parents of children with insomnia can have access to this intervention.

The BNBD intervention is protected by a trademark registered under the Canadian Intellectual Property Office. The Industry Liaison and Innovation Office with Research Services at Dalhousie University has executed an interinstitutional agreement for the BNBD study, which outlines and governs intellectual property (IP) rights, commercialization, and confidentiality and publication agreements between Dalhousie University and the partnering institutions: the Hôpital Rivière-des-Prairies, the Hospital for Sick Children, McGill University, University of Alberta, University of British Columbia, Université de Montreal, University of Toronto, and Western University. The IP relating to the session content remain with Dalhousie University and are licensed for use to the industry partner. The IP relating to the proprietary platform used to host the internet-based BNBD Intervention remains the property of the industry partner. Any IP that is jointly developed throughout the commercialization process has shared ownership.

### Conclusions and Significance

This research addresses significant public health issues through knowledge creation and translation to achieve direct benefits for the health and well-being of Canadian children and parents. This study aligns with the recognized need to more rapidly transfer new scientific knowledge to improve patient care and population health and targets the validation of new treatment delivery models to increase availability of effective treatment resources. This research is novel for Canadian and international pediatric health services and has the potential to have a direct and significant impact both in Canada and abroad.

## Appendix

Multimedia Appendix 1 Description of Measures.

Multimedia Appendix 2 CONSORT-EHEALTH checklist (V 1.6.1).

## Abbreviations

BNBD: Better Nights, Better Days
CONSORT: Consolidated Standards of Reporting Trials
eHealth: electronic health
HRQ: health-related questionnaire
IP: intellectual property
MID: minimal important difference
RCI: reliable change index
RCT: randomized controlled trial
