# Supplementary material for: Evaluation of an Internet-Based Behavioral Intervention to Improve Psychosocial Health Outcomes in Children With Insomnia (Better Nights, Better Days): Protocol for a Randomized Controlled Trial
Source: JMIR Res Protoc. 2018 Mar 26;7(3):e76. doi: 10.2196/resprot.8348 (PMC5891669; doi:10.2196/resprot.8348)
Supplement: Multimedia Appendix 1 [file resprot_v7i3e76_app1.pdf]

MULTIMEDIA APPENDIX:

Multimedia Appendix 1. Description of Measures

Screening Measure

Screening Questionnaire (SQ)

The author-made SQ is a screening instrument comprised of 12 items to assess whether participants meet study inclusion criteria and do not meet any of the exclusion criteria. A specific item addressing co-sleeping is modelled conceptually on Ramos and colleagues' [63] study examining parental perceptions of sleep problems. The questionnaire requires approximately 5 minutes to complete.

Eligibility Measures

Behavioural Insomnia Questionnaire (BIQ)

The BIQ is adapted by the authors based on the insomnia diagnosis criteria for children reported by Anders and Dahl [62]. The BIQ is comprised of 4 items to assess sleep onset disturbance and 4 items to assess night waking dyssomnia. An additional 9 items are included to capture sleep habits modelled from the ICSD-3/DSM-5 insomnia criteria. An evidence-based review [74] reported that the criteria, identified by Anders and Dahl, is one of few well-reported scales/assessments to evaluate insomnia symptoms in children. Only sleep onset items 1- 4 are used to assess eligibility in this study. Children are determined to meet sleep onset disturbance criteria if two of the following three conditions are fulfilled for at least one month: 1) more than three parental reunions occur per night for children ages 12-14 months, or more than two reunions for children older than 24 months; 2) sleep onset latency is greater than 30 minutes for children ages 12-24 months, or greater than 20 minutes for children older than 24 months; and 3) parental presence is required for sleep onset for two or more nights per week. The BIQ requires approximately 5 minutes to complete.

Pediatric Sleep Questionnaire (PSQ)

The PSQ developed by Chervin and colleagues [64] is a 22-item questionnaire which measures features of the presence of pediatric sleep related breathing disorders (SRBD), including snoring, daytime sleepiness, and behavioural disturbance. Response options to each item are 1 "Yes," 0 "No," or excluded "Don't know." The PSQ includes 3 subscales: snoring (4 items), sleepiness (4 items), and inattentive/hyperactivity behaviour (6 items). Responses to all non-excluded items are averaged to obtain a total score between 0.00 and 1.00. Scores with a cut off value of 0.33 indicate the presence of an SRBD. The PSQ demonstrates strong association with diagnosis of an SRBD ($P<0.0001$); diagnosis is also reported to be strongly associated across the 3 subscales [74]. The PSQ has been validated for use in children aged 2-18 years, and yields a sensitivity of (α=.81-.85), a specificity of (α=.87) and correct diagnostic classification for 85-86% of subjects. It also demonstrates good internal consistency (α=.89) and good test-retest stability (r=.92) [63]. The average of the first 6 items of the PSQ related to snoring and sleep disrupted breathing are used to assess eligibility criteria in this study. A score of 0.33 and greater does not meet inclusion criteria. The PSQ requires approximately 5 minutes to complete.

Health Related Questionnaire (HRQ)
The author-made HRQ is a 24-item questionnaire designed to identify the severity of parent-reported significant physical/mental health/sleep disorders. If a parent reports that their child has a diagnosed physical/mental health/sleep disorder, the HRQ is reviewed by a subcommittee composed of three BNBD co-investigators who determine eligibility based on clinical criteria. The HRQ requires approximately 5 minutes to complete.

Single Item Literacy Screen (SILS)
The SILS is a single-item questionnaire developed by Morris and colleagues [65] to assess functional literacy by asking potential participants whether they require help to read printed health-related material. Responses are rated on a 5-point scale, where 1 means "Never" and 5 means "Always." The SILS has been validated for sensitivity (54%) and specificity (83%) with an area under the Receiver Operating Characteristics Curve (ROC) of 0.73 [73]. Scores of 1 and 2 meet inclusion criteria for this study. The SILS requires approximately 1 to 2 minutes to complete.

Posteligibility Measures

Identifying Information Questionnaire (IIQ)
This author-made IIQ is designed to collect participant's mailing address and telephone number for the purpose of couriering study materials and communicating with parents. This information is collected separately from study data that could potentially identify the participant. The IIQ requires approximately 1 minute to complete.

Demographic Questionnaire (DQ)
The DQ is a 29-item questionnaire, adapted by the authors based on the format of the Canadian Consensus and the National Longitudinal Survey of Children and Youth [75]. The DQ is used to collect demographic and socio-economic information about the participant, the spouse/partner of the participant, and the child who is the focus of the intervention. The data collected is used to describe the sample and determine the representativeness of the sample from the Canadian population. The DQ requires approximately 5 minutes to complete.

Child Sleep/Insomnia Outcome Measures

Tayside Children's Sleep Questionnaire (TCSQ)
The TCSQ, developed by McGreavy and colleagues [76], consists of 10 items used to measure the presence of a disorder of initiating and maintaining sleep (DIMS) in children aged 1-5 years. The score of the first 9 items is calculated to assess insomnia using a 5-point frequency-scale anchored from 0 "Behaviour never occurs" to 4 "Behaviour occurs every night," while the final item assesses parental perception of the child's sleep problems. The TCSQ scale includes 3 subscales: initial settling; nighttime disruption; and early morning arousal. The TCSQ has been validated for its use to accurately measure sleep problems in children aged 1-5 years, and demonstrates a high internal consistency of (α=0.85) with item-total correlations of (r=.30 – .72) [76]. The total score assessed in this study is calculated by summing the first 9 responses, which can range from 0 to 36. A score of greater than 8.0 suggests the presence of DIMS. The TCSQ requires approximately 5 minutes to complete.

Sleep Disturbance Scale for Children (SDSC)

The SDSC, developed by Bruni and colleagues [77], is a 27-item instrument used to categorize sleep disorders in children aged 6-10 years. Items are rated on a 5-point Likert-scale. The SDSC scale includes five subdomains of sleep problems: disorders of initiation and maintenance of sleep, sleep disordered breathing, disorders of arousal, sleep-wake transition disorder, disorders of excessive somnolence, and sleep hyperhidrosis. The SDSC has been validated for children aged 6-10 years with high sensitivity (α=.89), and specificity (0.74%), and reliability (α=.79) coefficients. The SDSC demonstrates good reliability (α=.71) for subjects with sleep disorders and good total test-retest reliability (α=.71) [74]. Reliability ranges between (α=.21-.66) for single items [77]. A T-score is calculated for each subdomain and a total SDSC score is generated for the purposes of this study. The SDSC requires approximately 5 minutes to complete.

Child Daytime Functioning-Psychosocial/Physical Health Outcome Measures

Pediatric Quality of Life (Peds-QL)

The Peds-QL developed by Varni and colleagues [78] is a 23-item questionnaire which measures health related quality of life (HRQoL) in children and adolescents. Three parent-report versions are used depending on the age of the participant's child (Peds-QL for ages 2-4, 5-7, or 8-12) to accommodate the age range of the study sample. The Peds-QL uses a 5-point response scale anchored from 0 "Never" to 4 "Almost always." HRQoL is measured across 4 domains: physical functioning subscale (8 items), emotional functioning (5 items), social functioning (5 items) and school functioning (5 items). The Peds-QL yields adequate psychometric properties for physical health and psychosocial health, and has demonstrated reliability, validity, sensitivity and responsiveness for child self-report for ages 5-18 years and parent proxy report for ages 2-18 years. The validation study [79] identified a value approximately 1 SD below the populations mean for the Peds-QL total score (self-report 69.7, parent proxy report 65.4) as a threshold for an at-risk status for impaired HRQoL relative to the population sample. Items are reverse-scored and linearly transformed to a 0-100 scale. Higher scores reflect greater HRQoL. Subscale scores are calculated as the sum of items over the number of items answered. In addition to the four subscales, two summary scores are computed; the physical health summary score (8 items) is the same as the physical functioning score, and the physical health summary score (15 items) is the sum of items divided by the number of items answered in the emotional, social, and school functioning scales. The cut-off score assigned to physical health is any value 1 SD below the population sample mean (M=72.98), and this is also applied to psychosocial health (M=66.0) [78]. For the purposes of this study, the total PedsQL score and two summary scores are utilized. The Peds-QL requires approximately 5 minutes to complete.

Child Behaviour Checklist (CBCL/1 ½ -5, CBCL/6-18)

The CBCL developed by Achenbach and Rescorla [80] is designed to assess children's adaptive functioning and behavioural problems. Two parent-report versions are used to accommodate the age-range of the sample; the 100-item CBCL/1 ½ - 5 is validated for parents of children aged 1 to 5 years old [82], while the 113-item CBCL/6-18 is validated for parents of children aged 6 to 18 years old [83]. Parents are asked to rate the frequency with which their child displays a series of behaviours on a 3-point Likert-scale with response options ranging from 0 "Not true" to 2 "Very true or often true." The CBCL/1 ½ -5 and the CBCL/6-18 both include the following 7 subscales: withdrawn, somatic complaints, anxious/depressed, social problems, attention problems, delinquent/rule-breaking behaviour, and aggressive behaviour. The CBCL/6-18 also

includes the subscale thought problems. Subscale scores are further combined into two higher order factors: “internalizing” (withdrawn, somatic complaints, and anxious/depressed behaviour) and “externalizing” (delinquent/rule-breaking behaviour and aggressive behaviour). Both the CBCL/1½ -5 and CBCL/6-18 demonstrate high test-retest reliability for total scores, (r= .90) [82], and (r= .92) and (α= .97) respectively [83]. The CBCL/6-18 yields high reliability coefficients from 0.71 (somatic problems items) to 0.89 (conduct problems items), and strong convergent validity across DSM anxiety and affect oriented criteria [81]. For the purposes of this study, to assess clinical significance, scores are summed for each subscale and compared to a normative sample to obtain a T-score to indicate whether the child’s behaviour falls within “normal” limits, “borderline clinical” limits, or “clinical.” The CBCL requires approximately 10 minutes to complete.

Caregiver-Teacher Report Form for Ages 1 ½ - 5 (C-TRF) and Teacher Report Form (TRF) for Ages 6-18
The TRF developed by Achenbach and Rescorla [80] measures children’s adaptive functioning and behavioural problems, collected from teachers and caregivers. Two teacher-report versions are used to accommodate the age-range of the study sample; the 100-item C-TRF is appropriate for teachers of children ages 1 to 5 years [82], while the 113-item TRF is validated for teachers of youth ages 6-18 years [83]. Items are measured on a 3-point Likert-scale anchored from 0 “Not true” to 2 “Very true or often true,” with textboxes provided for qualitative feedback on certain items. The TRF and C-TRF both include the following seven subscales: withdrawn, somatic complaints, anxious/depressed, social problems, attention problems, delinquent/rule-breaking behaviour and aggressive behaviour. The TRF also includes the subscale thought problems. Subscale scores are further combined into two higher order factors: “internalizing” (withdrawal, somatic complaints, and anxious/depressed behaviour) and “externalizing” (delinquent/rule-breaking behaviour and aggressive behaviour). The C-TRF and TRF demonstrate sound psychometric properties. The C-TRF evidences high test-retest reliability (r=.88) [82], while the TRF demonstrates an average test-retest reliability of (r=.86), and good max internal consistency (α=.97) [83]. Used in conjunction with the CBCL, the C-TRF and TRF has good cross-informant agreement, (r=.72) and (r=.55) respectively [80, 82, 83]. To assess clinical significance, scores are summed for each subscale and compared to a normative sample to obtain a T-score to indicate whether the child’s behaviour falls within “normal” limits, “borderline clinical” limits, or “clinical”. These are optional measures which participants may forward to their child’s teacher used as external validation of findings based on parent reports. The C-TRF and TRF require approximately 10 minutes to complete.

Parent Functioning/Psychosocial Health Outcome Measures

Single Item Fatigue Impact Scale (SIFIS)
The SILS is adapted by co-author Reid (co-investigator on this study) from Chan and colleagues’ [84] 40-item Fatigue Impact Scale. The SILS consists of a single item that measures the severity of the impact of fatigue on parents. Responses are measured on a Likert-scale anchored from 0 “None” to 10 “A severely disabling effect.” Validity and reliability of the single item adapted questionnaire has not been reported; however, analysis of the 40-item Fatigue Impact Scale demonstrates high internal consistency (α=.91), adequate correlations (r=.60-.77) between each item’s rating and the summed ordinal ratings of the remaining items, and high construct validity and responsiveness [85]. The SIFIS requires approximately 1 to 2 minutes to complete.

Depression, Anxiety and Stress Scales (DASS-21)

Developed by Lovibond and colleagues [86], the 21-item DASS-21 evaluates self-reported distress by asking parents to indicate to what extent they have experienced certain mental health disorder symptoms in the past month. Items are anchored from 0 “Did not apply to me at all” to 3 “Applied to me very much or most of the time.” Items are measured across three subscales: depression, anxiety, and stress, which are combined to obtain a composite measure of general stress [87]. The DASS-21 demonstrates strong internal consistency with alpha values (α=.91) for stress subscale, (α=.87) for anxiety subscale, and (α=.94) for the depression subscale [88], as well as high sensitivity to the effects of parent-focused interventions [89]. The general stress composite score is the variable assessed in this study. The DASS-21 requires approximately 5 to 10 minutes to complete.

Parenting Scale (PS)

The 30-item self-report PS developed by Arnold and colleagues [90] is a measure of parental discipline strategies. Parents identify on a 7-point Likert visual analogue scale to what extent they engage in certain parenting practices. Each item receives a 1-7 score, where 7 is the "ineffective" end of the item. The PS assesses dysfunctional parenting across three stable factors: laxness, over-reactivity, and verbosity. The scale demonstrates good internal consistency (α =.84 to α =.87) [90, 91]. The average of responses on all items is calculated to provide a total score. To compute each factor score, an average of all items in that subscale is calculated. Both the total score and subscale scores are assessed in this study. The PS requires approximately 5 minutes to complete.

Measures for Exploratory Analyses

Children’s Physical Activity Index (CPAI)

The 5-item CPAI is adapted by the authors from the Children’s Physical Activity Questionnaire from the Canadian Health Measures Survey [92] to measure children’s level of physical activity. The questionnaire was developed for use in children aged 3-11 years. Participants are presented with 5-8 response options to identify the frequency of their child’s engagement in an activity. Each item receives a point value ranging from 1-8. The total score analyzed in this study is calculated by summing all items. The CPAI requires approximately 5 minutes to complete.

Body Mass Index (BMI)

Adapted by the authors from the Dieticians of Canada BMI calculator [93] for children and adolescents, the BMI measure is used as a measure of obesity of the participant’s child. BMI, rather than other measures of healthy weight, has been selected because the Canadian Paediatric Society, the College of Family Physicians of Canada, and Community Health Nurses Association of Canada recommend the use of the World Health Organization growth charts for growth monitoring of children and adolescents aged 2-19 years, which are based on BMI [94, 95]. Participants are instructed with a visual diagram, and are encouraged to use a scale and a measuring tape, which is couriered to them in their study package, to obtain accurate measurements for the height, weight and waist circumference of their child. BMI and percentile charts used in this study are calculated using the Dieticians of Canada BMI calculator. Scores that fall between the 3rd percentile and 84th percentile are considered to be within healthy weight for children between the ages 1-19 years. The BMI requires approximately 5 to 10 minutes to complete.

Parents Rating of Clinically Significant Improvement (PRCSI)
The author-made PRCSI is modelled conceptually on the theories reported by Montgomery and colleagues [96] to assess clinically significant improvement using analogue-scale parental-reports. The PRCSI contains 3 items to capture parent's satisfaction with the BNBD intervention. During baseline assessments, participants are asked to rate 2 items addressing: 1) what percentage improvement in their child's sleep problems they would be satisfied with, with responses anchored from 0%, "Not at all improved," to 100%, "Completely improved"; and 2) how helpful they anticipate this treatment program would be in treating their child's sleep problems, on a scale of 0% "Not at all helpful," to 100%, "Extremely helpful". During follow-up assessments, participants indicate how much their child's sleep problems have improved since they began the program. Participants are directed to select a percentage from 0% "Not at all improved" to 100% "Completely improved." Participants who wish to discontinue use of the intervention are also directed to complete the PRCSI and an additional open-ended question to ascertain why they have decided to not continue. The PRCSI requires 1 to 2 minutes to complete.

Treatment Utilization (TU)
The TU questionnaire developed by Reid as part of the Ontario Child Health Study [97] measures the extent to which parents utilize treatment resources. The TU includes 27 items to assess resource utilization across 8 categories: family physician, pediatrician, other person or place for regular health care, urgent care and emergency room, specialized health services, cost, medications, and other sources of help or advice. For most items, parents select "yes" or "no" to indicate what treatment resources they accessed. Parents elaborate on certain items to indicate what specific resources they have utilized. The TU is a predictor of treatment success and responses are used to assess the need for treatment intervention. The TU requires approximately 5 minutes to complete.

Willingness to Pay (WP)
The WP is an author-made questionnaire used to measure participant perception of the value of the intervention. The 7-item questionnaire asks participants how much they would be willing to pay for the intervention, and how much they would be willing to pay for additional treatment resources. Responses are used to assess the potential for commercialization and identify additional resources that could supplement the intervention. The WP takes approximately 5 minutes to complete.

Barriers to Treatment Participation Scale (BTPS)
The BTPS is a 10-item questionnaire adapted by Cunningham and Wuthrich [98] from the 44-item BTPS developed by Kazdin and colleagues [99] to assess parents' perceptions of barriers to participation in their children's treatment, treatment demands or issues, and relevance of treatment. Items are anchored on a 5-point Likert-scale that ranges from 1 "Never a problem" to 5 "Very often a problem." In addition, two author-made items are presented to gather information about contextual situations that could impact treatment participation. Response options are "yes" or "no," and participants can elaborate on their responses in text-box format. The BTPS demonstrates high internal consistency for both coefficient alpha and the Spearman-Brown coefficient (0.86) [99]. Items are summed to calculate the total score analyzed in this study, which reflects perceived barriers to participation in treatment. This questionnaire requires approximately 5 minutes to complete.

Client Satisfaction Questionnaire (CSQ-8)

The CSQ-8 developed by Larsen and colleagues [100] is widely used in primary care and mental health treatment to measure participant satisfaction with services received. The 8-item measure asks parents to rate the services they received through the BNBD intervention on a 4-point Likert-scale. Participants can also elaborate and provide any additional feedback on the intervention in text-box format. Internal consistency for the CSQ-8 is reported with alphas ranging from .83 to .93 [101]. An overall score for the CQS-8 is produced by summing all item responses producing scores ranging from 8 to 32, with higher values indicating higher satisfaction. The CSQ-8 takes approximately 5 to 7 minutes to complete.

Readiness for Change (RC)

The RC questionnaire developed by Reid and colleagues [102] based on the trans-theoretical model of change conceptualized for psychotherapy by Vyver [103] is administered to assesses whether participants are mindful and willing to adjust their parenting practices. The RC consists of 9 items on a 6-point Likert-scale ranging from “Strongly disagree” to “Strongly agree.” This scale consists of 5 primary domains: pre-contemplation, contemplation, decision making, action, and maintenance. Several studies have utilized the RC in the context of parenting successfully to predict parent commitment to treatment [102-104]. Parent responses are aggregated into “non-action” (pre-contemplation, contemplation, decision making) or “action” (action, or maintenance) and assessed as a predictor of treatment engagement. The RC takes approximately 5 minutes to complete.

Bedtime Routines Questionnaire (BRQ)

The BRQ developed by Henderson and Jordan [105] is a 31-item parent-report measure of children’s bedtime routine activities, specific to the consistency of the child’s weekday and weekend bedtime routines, reactivity to changes in the routine, and frequency of adaptive and maladaptive activities before bedtime. The BRQ consists of 3 scales: consistency (10 items), reactivity (5 items), and activities (16 items). Consistency and activities items are scored on a 5-point Likert scale ranging from 1 “Almost never” to 5 “Nearly always.” Reactivity items are scored from 1 “Not at all” to 5 “Extremely.” The BRQ has been validated for use in children aged 2-8 years and has an acceptable to excellent internal consistency coefficient of (α=.69) to (α=.90) for each primary scale calculated from an (n=222). Univariate ANCOVAs examining group differences between good and poor sleepers has revealed group differences on all four BRQ scales, $F(3, 88) = 2.74$ to 9.87, $p<.05$, demonstrating good discriminative validity [105]. Scores are calculated for each scale by summing items, with greater scores indicating great routine consistency, reactivity to changes, and more frequent activities. The consistency score is the variable assessed in this study as a predictor for treatment outcome; increased agreement with the use of limit setting strategies and rewards relates to better outcomes in children’s sleep. The BRQ takes approximately 5 minutes to complete.
